# Small Molecule Immunosensing Using Surface Plasmon Resonance

**DOI:** 10.3390/s100807323

**Published:** 2010-08-04

**Authors:** John Mitchell

**Affiliations:** Bioengineering Technologies Group, The New Zealand Institute for Plant and Food Research Ltd, East Street, Hamilton 3214, New Zealand; E-Mail: John.Mitchell@plantandfood.co.nz; Tel.: +64-7-959-4472; Fax: +64-7-959-4431

**Keywords:** immunosensor, surface plasmon resonance (SPR), small molecule, steroid, toxin, conjugation

## Abstract

Surface plasmon resonance (SPR) biosensors utilize refractive index changes to sensitively detect mass changes at noble metal sensor surface interfaces. As such, they have been extensively applied to immunoassays of large molecules, where their high mass and use of sandwich immunoassay formats can result in excellent sensitivity. Small molecule immunosensing using SPR is more challenging. It requires antibodies or high-mass or noble metal labels to provide the required signal for ultrasensitive assays. Also, it can suffer from steric hindrance between the small antigen and large antibodies. However, new studies are increasingly meeting these and other challenges to offer highly sensitive small molecule immunosensor technologies through careful consideration of sensor interface design and signal enhancement. This review examines the application of SPR transduction technologies to small molecule immunoassays directed to different classes of small molecule antigens, including the steroid hormones, toxins, drugs and explosives residues. Also considered are the matrix effects resulting from measurement in chemically complex samples, the construction of stable sensor surfaces and the development of multiplexed assays capable of detecting several compounds at once. Assay design approaches are discussed and related to the sensitivities obtained.

## 1. Introduction

**Figure 1.**
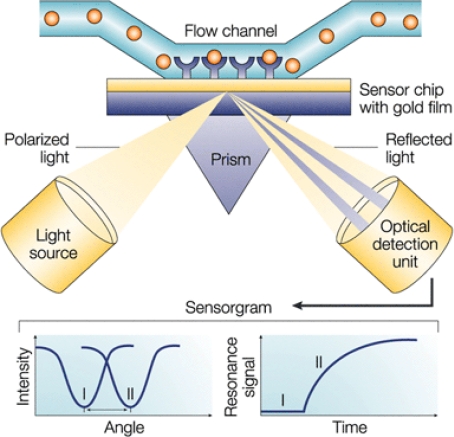
A schematic of the conventional Kretschmann optical configuration for SPR biosensing and the associated angle shift and sensorgram plot of resonance signal change with time [2]. Reprinted by permission from Macmillan Publishers Ltd: *Nat. Rev. Drug Discov.* **2002**, *1*, 515–528 copyright 2002; http://www.nature.com/nrd/index.html.

Surface plasmon resonance (SPR) is an opto-electronic phenomenon that occurs when a photon of light is incident upon a noble metal surface such as gold or silver [1]. When the wavelength of the photon equals the resonance wavelength of the metal, then the photon couples with the surface and induces the electrons in the metal surface to move as a single electrical entity called a plasmon. This oscillation of electrons sets up an electromagnetic field that exponentially decays out from the metal surface, with significant electrical field strength typically occurring within 300 nm of the surface. When molecules with sufficient mass bind to the surface within the range of the electric field, they perturb the plasmon and change the resonance wavelength. When dealing with a fixed planar surface, this is seen as a shift in the resonance angle of the incoming photons. These shifts essentially reflect minute refractive index changes on the surface and so can be used to very sensitively detect the binding of mass to the surface, typically down to a change of less than 1 × 10^−6^ refractive index units (RIU) for more sophisticated instruments (Figure 1). Refractive index is a ratio that changes from 1.0003 in air to 1.33 in water. Resonance units (RU) are often used to quantify refractive index changes in SPR biosensors, with 1 RU = 1 × 10^−6^ RIU, and so are used as units describing SPR signal strength.

**Figure 2.**
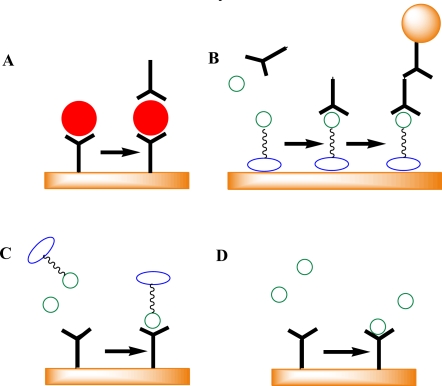
A schematic of some typical SPR immunosensor formats. **A.** Sandwich immunoassay for large molecules. **B.** Protein conjugate immobilized indirect inhibition immunoassay (can also link *via* self-assembled monolayers (SAMs) or carboxymethyl dextran polymers) with optional secondary antibody-gold nanoparticle labeling in a second step. **C.** Protein-labeled inhibition immunoassay. **D.** Direct small molecule immunoassay.

Immunoassays involve the binding of an antibody to its target antigen, and so the antibody, being a high mass (approximately 150 kDa) protein, can act as the signal generator in SPR transduction. Immunoassays therefore naturally lend themselves to SPR biosensing, and this is particularly the case for large molecule antigens (Mr > 2 kDa) which can be assayed in sandwich immunoassay formats. A sandwich immunoassay format would involve immobilizing antibodies onto the sensor surface, utilizing chemistries that include functionalized self-assembled monolayers (SAMs) [3], polymer coatings [4] and proteins such as protein A that orient antibodies on sensor surfaces [5]. The high mass antigen can then bind the immobilized antibody directly, thus generating biosensor signal and this signal can be further enhanced by binding a secondary antibody that recognizes another epitope on the antigen (Figure 2A). The design of large molecule immunoassays using SPR is now a very mature field of research.

Small molecule antigens, however, pose challenges not encountered with large molecules. The foremost of these is that the antigen itself can not generate very much SPR signal, given its small mass. There are some reports of kinetics studies and assays using direct detection of small molecule targets but these generally suffer from low signal and poor sensitivity (Figure 2D). To obtain optimal assay sensitivity the antigen is therefore either labeled with a high mass label and used in competition with un-labeled sample antigen for binding to the surface in a competitive immunoassay, or the small molecule antigen is conjugated to the sensor surface and primary antibody is mixed with sample containing free antigen and the mixture is passed over the sensor surface (Figures 2B and C). In this case the mass is provided by the primary antibody and signal can be further enhanced by use of secondary antibodies either with or without conjugation to gold nanoparticles [4] (Figure 2B). In either case, the sensor signal is inversely proportional to the concentration of the antigen free in solution. Gold nanoparticles can provide signal enhancement both through their high mass and through cooperative plasmon enhancement by coupling between the localized plasmon field of the nanoparticle and the surface plasmon field of the gold sensor surface [6]. Small molecule assay formats require very careful design of the surface chemistry and the labeling employed so as to ensure optimal sensitivity.

Another critical concern with small molecule immunoassay using SPR is the potential for steric hindrance of the binding between antigen and antibody when there is either a large label proximal to the antigen or where the antigen is bound to the sensor surface. Careful use of appropriate linker chemistry can help mitigate these constraints and improve binding signal and sensitivity. The stability of the immunobiosensor surface to the harsh solutions used to regenerate and re-use the surfaces ready for another assay must also be considered, particularly for high-throughput applications. The use of high quality antibodies with high affinity towards the target compound are also important to achieving high SPR sensor signal and low limits of detection (LOD). If the antibody binds the analyte strongly then smaller concentrations of the analyte may be needed to inhibit antibody binding to the sensor surface, thus reducing LOD. Typically, antibodies with affinity constants of 1–10 × 10^9^ L/mol are desirable. Another major consideration is the resistance of the surface to biofouling caused by non-specific binding from high mass contaminants in complex real-world samples. These can both disrupt antigen/antibody binding and deposit high mass on the surface, potentially distorting immunoassay signals.

This review will examine the general principles and parameters of small molecule immunoassays and how others have designed their immunosensor systems using SPR transduction. Specific classes of target small molecule antigens of particular interest are then investigated, namely steroids, toxins and food components, drugs and explosive residues, before some general comments are made on possible future directions in small molecule SPR immunosensor research. A summary of some SPR immunoassays of small molecules is given in Table 1.

## 2. General Principles and Immunosensor Assay Formats

When developing small molecule immunobiosensors for use with SPR transduction, one must first consider the structure and assembly of the sensing surface. The small molecule antigen can be immobilized to the gold surface *via* the commonly used carboxymethyl dextran polymer layers. These layers are functionalized with carboxylic acid groups that allow covalent attachment of antigen or antigen derivatives containing an amino group through the formation of an amide bond. Steroid hormones have been conjugated using this technique in a convenient *in-situ* flow-through immobilization technique within the biosensor by attaching a linker at a point on the antigen distant from existing functional groups [4]. The resulting functionalized surfaces can withstand up to more than 1,100 binding and regeneration cycles [4]. They use hydrophilic oligoethylene glycol (OEG) linkers which can project the antigen into the aqueous fluid stream and allow for optimal antibody binding [4,7]. Binding is also optimized through careful selection of the position of conjugation on the steroid so that linkers are attached distant from existing functional groups [7].

**Figure 3.**
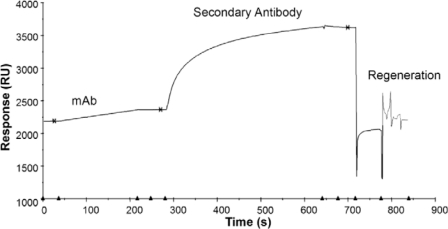
A sensorgram of the primary monoclonal antibody (mAb) binding response to a progesterone-immobilized SPR sensor surface and enhancement of binding signal with secondary antibody followed by regeneration. Reprinted from reference [4], with permission from Elsevier. Copyright 2005.

Another key consideration is the method of signal generation. With antigen-immobilized formats, the primary antibody can provide the signal but studies using steroid antigens have also employed secondary antibodies to further enhance the signal strength with enhancements of 6–8 times the primary antibody binding signal [4] (Figure 3). In addition to this enhancement approach, gold nanoparticles have been used to further enhance signal by adding more bound mass and through cooperative plasmon coupling. Gold nanoparticles concentrate a high mass into a small volume. Their surfaces allow convenient formation of coordinate bonds with thiol functional groups and so they can be easily conjugated to biomolecules as signal enhancement labels. The diameters of the nanoparticles can also be easily adjusted between 1–100 nm as required. The noble metal nanoparticles also undergo SPR themselves and so when they approach a gold SPR sensing surface their plasmon modes can couple with those of the surface and produce a large shift in refractive index and thus an enhancement of SPR signal much larger than that expected on the basis of high mass labeling alone [6]. This labeling approach has been widely applied to SPR sandwich immunoassays of large molecules but has only recently been extended to small molecules. When 25 nm gold nanoparticles conjugated to secondary antibodies were employed, enhancements of 13-fold were achieved for immunosensing of progesterone with a LOD of 8.6 pg/mL [4]. Protocols for the enhancement of small molecule SPR immunosensing have now been standardized and these approaches can provide a generic platform for small molecule immunosensing combining linker chemistry with nanoparticle enhancement [8]. The range of small molecule analytes to which gold nanoparticle enhancement has been applied is now growing and includes the steroid hormone metabolite estriol-16-glucuronide [9,10], the antibiotic chloramphenicol [11] and the neurotoxin ochratoxin A [12]. Consideration has also been given to the effects on SPR signal enhancement of the diameter of the nanoparticles [13] and their distance from the sensing surface [14].

**Figure 4.**
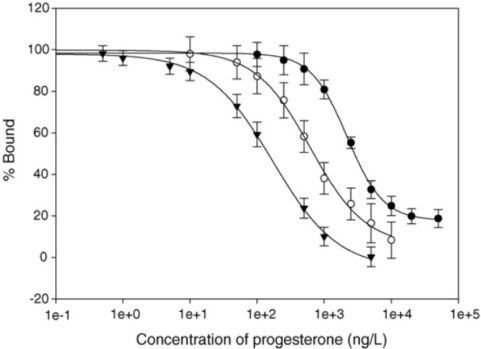
Standard curve plots for progesterone using a mixed SAM layer with protein-conjugate immobilization for SPR immunosensing using mAb only (•), secondary antibody enhancement (○) and 10 nm immunogold enhancement (▾). Reprinted from reference [3], with permission from Elsevier. Copyright 2007.

Similar enhancement techniques can also be employed with other types of surfaces such as thiol SAMs terminating in carboxylic acid groups [3] (Figure 4). This approach has been used in combination with protein conjugates of the antigen to construct sensor surfaces for detection of progesterone [3], chloramphenicol [11] and ochratoxin [12]. In these cases gold nanoparticles of 10 nm, 40 nm and 40 nm, respectively, were employed. Whilst SAMs allow the binding events to take place closer to the gold surface where the field strength is higher, they are typically less stable under regeneration than dextran surfaces [3].

An alternative format for analyzing binding interactions in small molecule SPR immunosensing is to immobilize antibody and examine binding of small molecule protein conjugates. This approach has been used for progesterone when examining the effects of intermediate linker length on antibody binding strength, where a linker of 18-atoms was found to give high binding signal [15].

Most SPR immunoassays for small molecules are conducted in a microfluidic environment. Mathematical models of the effect of various system parameters such as flow rate, antibody concentration, and density of binding sites on the surface have been developed to help optimize sensitivity [16]. Such modeling approaches have been applied to concentration gradient immunoassay where space and time-dependent binding is analyzed in a two-dimensional imaging SPR configuration with introduction of sample and antibody through ports in a T-shaped sensor [17]. This approach involves parallel streams of fluid, one containing analyte and the other antibody and analyzes the steady state gradient set up by interdiffusion between the fluid streams through binding to the immobilized antigen phenytoin [18].

Multiplexing assays in array formats is of increasing interest with small molecules, given the success of protein arrays [19]. SPR imaging is a format that utilizes the localized plasmon fields of specially patterned metal surfaces to detect SPR shifts spatially in two-dimensions. It is widely used in the study of protein binding interactions [20]. A photo-crosslinked small molecule platform has been successfully applied to detection of receptor binding to estrogens using SPR imaging [21]. Screening protein-protein interactions in a microarray format using scattered light under SPR conditions is emerging as an imaging SPR format [22] and may be adapted in future to small molecule immunoassays.

Another key consideration is the continued functioning of the sensor in the presence of complex sample matrices such as serum [23] and human saliva. There is also on-going interest in making SPR small molecule sensor systems portable, and this is particularly useful in environmental measurements requiring on-site, real-time analysis. Benzo[a]pyrene and 2-hydroxybiphenyl have been detected using an indirect competitive immunoassay where the SPR sensor system is confined to dimensions of 16 cm × 9 cm × 6 cm in a four flow-channel system [24].

Recently, interest has been building in alternatives to antibodies as binding agents in small molecule SPR formats. Aptamers (protein or oligonucleic acids that recognize specific target molecules) are being considered whereby binding between the aptamer and an immobilized partial complementary ss-DNA strand is disrupted by the presence of the small molecule target. This has been applied with gold nanoparticle labeling for detection of adenosine [25]. Such formats have shown detection performance down to 1 nM when used as an SPR biosensor for adenosine [26].

## 3. Steroids

Analysis of steroid hormones is of interest to scientists investigating human physiology, optimization of athletic training regimes, monitoring of reproductive cycles in humans and animals for artificial insemination and pregnancy planning, and in the diagnosis and treatment of hormonal disorders. SPR has been applied to the measurement of progesterone in milk for potential use in monitoring estrous cycles in cows [27]. This system immobilizes progesterone *ex-situ* by removing the carboxymethyl dextran coated gold sensing surface from the chip cover and dispensing onto it a solution of 3-carboxymethyloxime derivative outside the SPR instrument. It demonstrated a LOD of 3 ng/mL which was later improved to 0.4 ng/mL in cow’s milk [27,28]. This system uses significant dilution to overcome matrix effects [27,28], which can compromise the overall assay sensitivity. Estradiol, in both serum and seawater has been sensed using a combination of SPR and a coupled online in-tube solid-phase microextraction (SPME) system. This inhibition immunoassay gave an LOD of 170 pg/mL [29]. Estradiol has been measured in buffer-based assays using OEG covalent linker conjugation and LOD of 25 pg/mL were obtained when conjugation position is carefully considered [7]. Another report utilizes an estradiol-protein conjugate for immobilization and achieves inhibition at concentrations of about 0.6–30 ng/mL [30]. In serum, SPR has shown that sex hormone binding globulin can form aggregates that may impact on protein immunoassay performance [31]. Testosterone and estradiol have been derivatized with biotin and pre-incubated with streptavidin before immobilization on the sensor surface. Subsequently, antibody binding kinetics were examined and showed good correlation with quartz crystal microbalance (QCM) results [32].

**Figure 5.**
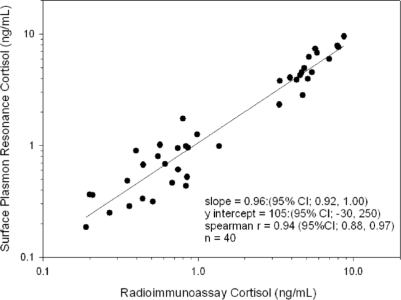
Correlation plot for salivary cortisol samples analyzed by an SPR biosensor and by radioimmunoassay for 40 samples, from reference [33]. Reproduced by permission of The Royal Society of Chemistry (RSC).

The highly sensitive detection of the steroid hormones cortisol and testosterone in human saliva is of great interest to physiologists studying the effects of exercise on athletic performance. It can potentially provide a non-invasive means of monitoring hormonal fluctuations with time and could also be used to help diagnosis of hormonal disorders such as Addison’s disease and Cushing’s syndrome. Utilizing the covalent immobilization and OEG linker technology developed for progesterone [4], it has been possible to construct highly sensitive SPR immunoassays for both cortisol [33] and testosterone [34] (Figure 5). LOD of 49 pg/mL for cortisol and 15.4 pg/mL for testosterone have been reported in stripped human saliva matrix [33,34]. The typical physiological range is 0.1–10 ng/mL for salivary cortisol and 29–290 pg/mL for salivary testosterone [35]. The cortisol immunosensor uses secondary antibody enhancement of SPR signal [33], whilst the testosterone assay uses secondary antibody-nanogold enhancement to achieve greater sensitivity [34]. Non-specific binding has been reduced in the case of cortisol by the use of a surfactant in the antibody diluents, which removes the need for chemical extraction or extensive pre-treatment of saliva samples [33]. Other groups have proposed the use of a combination of membrane filtration and an H-filter [36] to remove mucins and other high mass components in saliva or using a flow-filter arrangement [37] but this can add time, expense and complexity to the biosensor system [36] and risk repeated fouling of membranes. A cortisol-protein conjugate immobilized format has demonstrated an LOD of 1 ng/mL, insufficient to cover the full physiological range for salivary cortisol [37]. Subsequent work attempting direct immunoassay has demonstrated poor sensitivity, with reported linear detection regions ≥13 ng/mL for saliva and ≥9 ng/mL in urine [38]. Cortisol has also been used as a target antigen in the development of SPR immunosensors utilizing nanohole array substrates [39]. Such a set-up has the advantage of simple optics (they only need a simple UV-visible spectrophotometer) giving the potential for portable units to be constructed. Detection of small molecules such as the steroids in environmental samples is of increasing importance [40] as understanding is gained of their effects on freshwater and marine ecosystems, and so SPR has also been applied to environmental measurement of 17β-estradiol in seawater [41].

## 4. Toxins and Food Safety and Composition

One of the key areas of interest in small molecule SPR immunosensing is the detection of toxins in foods and beverages to comply with increasingly strict requirements for food safety testing [42–44]. The key requirements are highly sensitive detection in complex matrices with high throughput. Also of interest is the detection of bioactives and additives in foods [42]. Much of the work in this area is focused on veterinary drugs, shellfish toxins and antibiotic residues, with less work devoted to mycotoxins. SPR is increasingly being explored as a transduction platform for such assays [45] along with other biosensor techniques, such as quartz crystal microbalance technology and electrochemical transduction [46]. Detection of toxins on-site using portable SPR is also beginning to be investigated [47]. The antibiotics neomycin and gentamycin have been measured in a parallel format using an imaging SPR system from IBIS Technologies, in a competitive assay with antibiotic immobilized through its amino groups to a carboxymethylated dextran. This resulted in detection ranges in the 1–50 ng/mL region [48]. The poor sensitivity is likely due to the absence of a linker between the antigens and the surface, or conjugation through an existing functional group.

**Figure 6.**
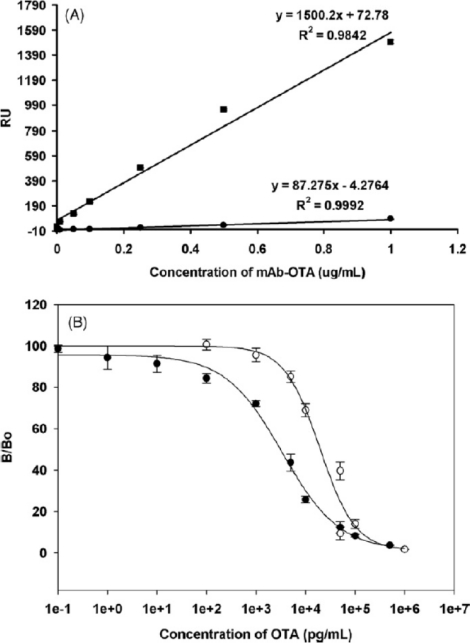
A: Plot of response (RU) v. primary antibody concentration for mAb only (•) and 40 nm immunogold enhancement (▪). B: Assay standard curves for mAb only format (○) and 40 nm immunogold enhanced format (•). Reprinted from reference [12], with permission from Elsevier. Copyright 2009.

Mycotoxin immunoassay using biosensors has attracted attention as a new technology for food safety screening [49]. Combined detection of T-2 and HT-2 toxins is possible using SPR by organic extraction from baby food, breakfast cereal and wheat. LOD of 25–26 μg/kg were obtained with CVs of 1.8–6.3% [45]. Fungal metabolites are often toxic, and an example is deoxynivalenol which can be conjugated to casein to coat an SPR biosensor surface for a competitive immunoassay [50]. These surfaces are reported to be stable for about 500 assay cycles but function in a high concentration range of 2.5–30 ng/mL [50]. Ochratoxin has been determined in cereals and wine using ochratoxin-OEG-ovalbumin protein conjugates on a SAM layer with 40 nm gold nanoparticle signal enhancement [12] (Figure 6). A radically different format for ochratoxin detection is based on hollow gold nano-sized balls coated in a dendritic thionine thin film for immobilization and sensing [51]. The technique was also tried in milk [51]. Staphylococcal enterotoxin can be detected in raw eggs using an antigen immobilized SPR competitive immunoassay which allows detection at 1–40 ng/mL from supernatant [52]. Aflatoxin B1 has been the subject of a protein conjugate-immobilized sensor format, with a polyclonal antibody and special regeneration conditions to overcome strong antibody binding [53]. In comparison, another study has favored using immobilized enzyme in an SPR format to detect aflatoxin [54].

Shellfish toxin detection is another major application that has been explored extensively using SPR immunosensing. Okadaic acid has been detected by organic extraction from mussels and scallops and demonstrated minimal matrix effects in a direct competitive assay format and with an LOD of 0.24 ng/mL in mussel extract, allowing detection below European regulatory limits of 160 μg/kg [55]. It has also been detected using an amine coupling method which has shown stability over more than 50 assay cycles, though the immobilization method used may suffer from steric hindrance of antibody binding [56]. Antibodies have been raised to domoic acid and immunoassays established in both competitive and displacement assay formats, with the competitive format giving an LOD of 3 ng/mL and correlating well to HPLC when applied to measurement in clams [57]. Another study has shown that domoic acid-immobilized surfaces can be stable for 800 assay cycles, with measurement complete within 10 min and with reported LOD in the ng/g region, below the EU action limit of 20 μg/g [58]. The binding interactions between domoic acid and polyclonal, monoclonal and recombinant antibodies have been examined using SPR formats with immobilized domoic acid [59]. A number of paralytic shellfish toxins have also been measured using SPR by employing a new saxitoxin polyclonal antibody and compared with ELISA and mass spectrometry techniques. The biosensor had a reported best LOD of 21.6 μg/100 g of shellfish compared to a European regulatory limit of 80 μg/100 g of shellfish [60]. Proteins of potential use in assays of saxitoxin have been screened using SPR by immobilizing the toxin *via* amine coupling onto the sensor surface [61].

**Figure 7.**
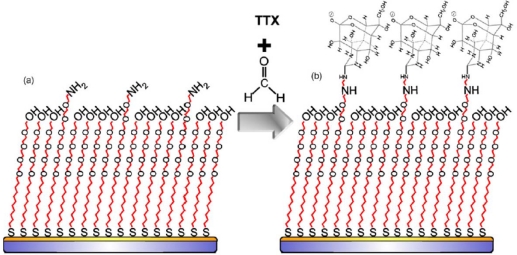
Immobilization of tetrodotoxin onto a mixed thiol SAM on an SPR gold surface. Reprinted from reference [64], with permission from Elsevier. Copyright 2008.

SPR has also been used to probe the binding interactions between proteins and toxins and to examine such effects as the interaction between the ABC ring structure of ciguatoxin and its specific antibody, revealing that the bulkiness and aromatic nature of the antigen was critical to specific antibody recognition [62]. Another study has immobilized ciguatoxin using a 3-butene-1,2-diol side chain attached to the A-ring and then examined antibody binding interactions [63]. Tetrodotoxin has been measured using a modified OEG SAM surface structure, which achieved minimization of non-specific binding and an LOD of 300 pg/mL [64] (Figure 7). Using this approach the ratios of the functionalized and blocking SAM thiol chains could be optimized to provide adequate spacing between immobilized antigen molecules to allow stronger antibody binding [64]. 2,4-D can be detected by using a protein conjugate [65,66] to immobilize the antigen and an indirect competitive assay format. The latest report indicates an LOD of 100 pg/mL using multiple flow channels [65]. An emerging related method is the use of total internal reflection ellipsometry, which may provide an alternative transduction approach for sensitive detection of small molecule toxins such as T-2 mycotoxin [67].

Determination of food composition using SPR immunosensing appears to have attracted much less attention than toxins detection, but investigators have measured compounds such as vitamin B-5, which was measured in a wide range of foods including reference samples, giving a LOD of 4.4 ng/mL using buffer extraction [68].

## 5. Drugs

Localized surface plasmon resonance (LSPR) has been used for the detection of the drug stanozolol using immobilized gold nanoparticles [69,70]. Here stanozolol-protein conjugate is electrostatically adsorbed onto the gold, and antibody binding to the surface is detected with an LOD of 0.7 ng/mL [70]. Often, detection of drug traces on surfaces is of interest, so a SensiQ^®^ SPR biosensor has been applied to detection of methamphetamine traces on ceramic tiles down to 25 ng/100 cm^2^ [71]. Using LSPR, it is possible to multiplex assays in an array and this has been applied to simultaneous detection of cocaine, ecstasy, heroin and amphetamine using a combination of antibodies and with antigen-protein conjugates immobilized on the array and allowing both SPR and ellipsometric sensorgram readouts [72]. The beta-agonist clenbuterol has been detected in urine and serum and the matrix effects involved have been investigated [73]. This study found that non-specific binding from urine was greatly influenced by salt concentration and pH and that ultracentrifugation was effective in combating non-specific binding for both urine and serum [73]. Another format detects chloramphenicol using a SAM/protein conjugate surface and gold signal enhancement [11]. Fenicol antibiotic residues in shrimp have been examined using organic extraction followed by detection of four different fenicols over two flow cells using a direct amine coupling to carboxymethylated dextran without linkers [74]. Chloramphenicol can be detected in milk using a BIAcore^™^ inhibition format involving amine coupling of the antigen to the sensor surface and using an antibody recognizing an epitope distant from the amine group and thus helping to improve antibody binding and sensitivity [75]. An LOD of 0.1 ng/mL is reported [75]. Warfarin has been examined by SPR, with the formation of a panel of warfarin-protein conjugates which were then used to raise a selection of anti-warfarin monoclonal antibodies [76]. These antibodies were then applied in inhibition assays of warfarin to determine the un-bound fraction in blood plasma ultrafiltrate [76]. This technique was compared with HPLC and gave a detection range of 4–250 ng/mL [76]. The sensor surfaces were produced by covalent amine coupling of 4′-aminowarfarin [76].

The study of binding interactions between antibodies and drug molecules is a rapidly emerging area where SPR’s biomolecular interaction analysis capability is being utilized to understand the kinetics of drug binding interactions. This field of fundamental biomedical research is too large to cover adequately in this review, but recent examples include the covalent immobilization of the drug panitumumab to a carboxymethylated BIAcore^™^ surface by amine coupling to assess its clinical immunogenicity [77]. In another example, single-chain fragment variable (scFv) antibody fragments are identified that can recognize a heroin metabolite without recognizing morphine. The SPR biosensor was used to determine binding affinities using a biotin-PEG linker to conjugate the metabolite to the surface [78].

**Figure 8.**
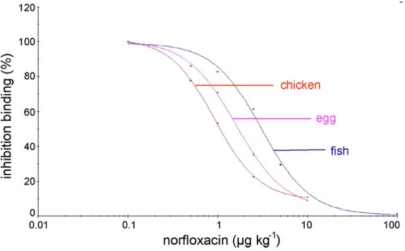
Assay standard curves for norfloxacin detection in chicken meat, egg and fish. Reprinted from reference [85], with permission from Elsevier. Copyright 2008.

In addition to the detection of drugs in humans and animals, the determination of antibiotic residues in foods, and beverages such as milk, is also of significant interest as researchers look for alternatives to centralized LC-MS technology [79,68]. Immunosensors are seen as a potential way forward to faster and more affordable analysis [80]. β-Lactams have been measured in milk using a SPR surface where the analyte is immobilized *via* an immunogenic interaction between H1 and its complement antibody and gives detection for penicillin G as low as 1–2 μg/kg [81]. Benzimidazole carbamate residues have been detected in milk also, this time using a liquid extraction/partition technique and immobilized antigen-protein conjugate, giving an LOD of 2.7 μg/kg [82]. Also detecting in milk, an imaging SPR system was developed to detect five different antibiotic residues in 10-fold diluted milk, with performance comparable to conventional SPR designs [83]. Taking multiplexing even further, 13 fluoroquinolone antibiotics have been detected in poultry, fish and eggs [84,85] (Figure 8) giving detection for norfloxacin of below 0.5–1.5 ng/g [84]. Fluoroquinolone SPR has been compared with LC-MS techniques for identification of contaminated samples and has performed well in this regard and demonstrated time and sensitivity advantages over more conventional microbiological techniques [86]. Flumequine, another fluoroquinolone, has been measured in the blood serum and muscle of broiler chickens using immobilized antigen and detected concentrations of 15–800 ng/mL [87]. SPR techniques can also be used in concert with more conventional LC-MS techniques to analyze fluoroquinolones. The SPR biosensor can serve to screen the antibiotics of interest and identify relevant fractions for further investigation with LC-MS [88]. In the case of penicillin G detection in milk, enzymes have been applied in place of antibodies but still using SPR transduction [89].

The antibiotic cephalexin was detected in the range of 244–3,900 ng/mL in milk using an antigen-protein conjugate immobilized regenerable surface with a polyclonal antibody [90,91]. The high detectable concentration range in the assay could be attributed, at least in part, to the lack of a spacer chain between the cephalexin and the carrier protein and conjugation through an existing functional group on the drug analyte. Nicarbazin is a coccidiostat two-drug combination used as an additive to chicken feed to prevent disease outbreaks. One of these drugs, 4,4-dinitrocarbanilide is detected in an SPR format that immobilizes a mimic of the drug on a carboxymethyl dextran surface for detection from liver and egg samples with LOD of 17–19 ng/g [92].

Some progress has been made in the development of prototype systems capable of high-throughput detection. Bile analysis for sulfamethazine and sulfadiazine has been reported, where up to eight samples can be simultaneously detected with a throughput of up to 650 samples per day with low false negative and false positive rates [93]. This prototype technology was also applied to measurement of clenbuterol and ethinylestradiol in urine and to sulfamethazine, sulfadiazine and enrofloxacin in milk [94]. An earlier report detailed a sulfamethazine immunoassay using SPR in animal urine with LOD of 5 ng/mL for use as a drug residue screening test [95]. This format interestingly uses anti-idiotypic antibodies immobilized on the sensor surface and detects sulfamethazine through the inhibition of monoclonal anti-sulfamethazine binding to the surface [95]. Sulfamethazine has also been detected in milk using covalent attachment of the antigen to a carboxymethyl dextran surface and trying two different antibodies, giving a best LOD of 1.7 μg/kg [96]. An earlier study reported detection down to <1 nM in milk [97]. Use of a secondary antibody to further enhance the signal in sulfamethazine immunoassay using SPR allowed reduction of the primary antibody concentration used [97].

BIAcore™ SPR detection has been applied to ivermectin determination in bovine liver using acetonitrile extractions and solid phase extraction (SPE) clean-up with LOD of 19.1 ng/g [98]. Streptomycin residue detection has been examined in an indirect inhibition assay format using protein conjugate immobilization and in a direct assay format. Both of these approaches led to poor detection performance, with IC_50_ of 10–20 ng/mL [99]. 7-Hydroxycoumarin (umbelliferone) can be detected in human serum using SPR transduction where an antigen-protein conjugate is immobilized on a BIAcore™ sensor chip measuring in the range of 0.5–80 μg/mL [100]. The buffer composition was developed to try to minimize non-specific binding [100].

## 6. Explosives Residues

SPR is beginning to be applied to detection of explosives residues [101], forming a growth area in SPR immunosensing. 2,4,6-Trinitrotoluene (TNT) has been detected using an indirect competitive format where a TNT analogue is immobilized to the sensor surface in the form of a 2,4,6-trinitrophenyl-keyhole limpet hemocyanin conjugate [102]. Various monoclonal and polyclonal antibodies were tested for their binding strengths, and a lowest LOD of 2 pg/mL was reported when using a polyclonal antibody raised to the same conjugate [102], showing that careful matching of the antibody and surface chemistry was central to obtaining good detection performance. TNT has also been assayed using a dendrimer-modified SPR surface [103]. In this format, a thiol SAM combined with a poly(amidoamine) (PAMAM) dendrimer provides the support structure for attachment of dinitrotoluene-keyhole limpet hemocyanin conjugate which serves as the immobilized antigen for competitive immunoassay of TNT using a monoclonal antibody and giving an LOD of 110 pg/mL on a regenerable surface [103]. Another study of TNT SPR immunosensing uses a mixed thiol SAM where some of the thiols act as blocking agents and others are functionalized for immobilization of the antigen [104]. Three TNT analogues are used for immobilization and their efficacy in the immunoassay is assessed. The surfaces are reported to exhibit low non-specific binding [104]. Irreversible gas-phase detection of TNT has also been reported using a dry surface [105]. Hexahydro-1,3,5-trinitro-1,3,5-triazine (RDX) has been detected by an interesting SPR sensor construction that involves cross-linking gold nanoparticles using electropolymerized bisaniline cross-linking in the presence of Kemp’s acid, which yields an imprinted nanocomposite that demonstrated a high binding affinity for the explosive analyte [106]. The dielectric properties of the composite changes as π-donor –acceptor complexes are formed, which alters the localized surface plasmons of the nanoparticles, giving a reported LOD of 12 fM [106]. An OEG functionalized surface has been used to detect 2,4-dinitrotoluene with SPR immunosensing, whereby the analyte is immobilized onto an OEG SAM and an indirect competitive immunoassay is set up using a specially prepared polyclonal antibody [107]. An LOD of 20 pg/mL was obtained and binding response maintained for more than 30 assay cycles [107].

## 4. Conclusions

The use of SPR transduction for the immunosensory detection of important small molecule analytes has expanded rapidly over recent years, with particular interest in developing routine testing methods for toxins in foods, and drug residues in both foods and biological fluids. Its use for sensitive detection of steroid hormones and explosive residues has now also been explored. From the research covered in this review, it is evident that, provided a good quality antibody is used, those studies that have most carefully examined the structure of the sensing surface have achieved the best results in terms of both sensitivity and surface stability. Some studies still use direct amine coupling to sensor surfaces without use of intermediate linkers and so may sacrifice sensitivity through steric obstruction of antibody binding. When factors such as linker lengths, conjugation techniques, protein and gold nanoparticle enhancement and regeneration stability are considered and optimized then highly sensitive small molecule immunosensor systems can be developed that can detect in the pg/mL concentration region and overcome the challenges of small analyte size and steric obstruction of binding interactions. In future, it seems likely that the range of small molecule analyte targets examined will expand and that even more attention will be devoted to sensor surface construction, both in terms of the chemical immobilization layers and the underlying metal substrate, particularly given the promise of LSPR techniques for multi-analyte detection. It is hoped that in time SPR immunosensing of small molecules will become routine in testing laboratories and that portable SPR transduction units will expand the range of applications of plasmonics where on-site and near real-time detection is crucial.

## Figures and Tables

**Table 1.** A summary of some SPR immunoassays of small molecules, giving the target analyte, the sample medium, reported limit of detection (LOD) and method of enhancement (if used). Note that researchers often use different methods for calculating LOD.

**Target**	**Medium**	**Reported LOD**	**Enhancement**	**Reference**
2,4,6-Trinitrotoluene	Buffer	2 pg/mL	none	[102]
2,4,6-Trinitrotoluene	Buffer	110 pg/mL	none	[103]
2,4,6-Trinitrotoluene	Buffer	1,000 pg/mL	none	[104]
2,4,6-Trinitrotoluene	Vapour	700 pg/mL	none	[105]
2,4-D	Buffer / river water	100 pg/mL	none	[65]
2,4-D	Buffer / river water	8 pg/mL	antibody / protein	[65]
2,4-Dinitrotoluene	Buffer	20 pg/mL	none	[107]
2-Hydroxybiphenyl	Buffer	100 pg/mL	none	[24]
7-Hydroxycoumarin	Serum	500,000 pg/mL	none	[100]
Aflatoxin B1	Buffer	3,000 pg/mL	none	[53]
Aflatoxin B1	Maize	970 pg/g	none	[54]
Amphetamine	Buffer	5,000 pg/mL	none	[72]
Benzimidazole carbamate	Milk extract	2,700 pg/g	none	[82]
Benzo[a]pyrene	Buffer	100 pg/mL	none	[24]
Cephalexin	Buffer	4,880 pg/mL	none	[90, 91]
Cephalexin	Milk	244,000 pg/mL	none	[90, 91]
Chloramphenicol	Shrimp extract	100 pg/mL	none	[74]
Chloramphenicol	Milk	100 pg/mL	secondary antibody	[75]
Chloramphenicol	Buffer	0.00074 pg/mL	40 nm gold	[11]
Cocaine	Buffer	2500 pg/mL	none	[72]
Cortisol	Saliva	49 pg/mL	secondary antibody	[33]
Cortisol	Buffer	360 pg/mL	none	[37]
Cortisol	Saliva	1,000 pg/mL	none	[37]
Cortisol	Saliva / Urine	2,000 pg/mL	none	[38]
Cortisone	Saliva / Urine	9,000 pg/mL	none	[38]
Deoxynivalenol	Wheat extract	2,500 pg/ml	none	[50]
Dihydrostreptamycin	Milk	20,000 pg/mL	none	[99]
Domoic acid	Clam extract	3,000 pg/mL	none	[57]
Domoic acid	Shellfish	> 1000 pg/g	none	[58]
Ecstasy	Buffer	5,000 pg/mL	none	[72]
Estradiol	Serum/Seawater	170 pg/mL	none	[29]
Estradiol	Buffer	25 pg/mL	secondary antibody	[7]
Estradiol	Buffer	600 pg/mL	none	[30]
Estradiol	Seawater	455 pg/mL	none	[41]
Estriol-16-glucruonide	Buffer	14 pg/mL	15 nm gold	[9]
Estriol-16-glucruonide	Urine	16 pg/mL	gold	[10]
Florefenicol	Shrimp extract	200 pg/mL	none	[74]
Florefenicol amine	Shrimp extract	250,000 pg/mL	none	[74]
Flumequine	Chicken serum	15,000 pg/mL	none	[87]
Flumequine	Chicken muscle	24,000 pg/g	none	[87]
Heroin	Buffer	500 pg/mL	none	[72]
Ivermectin	Bovine liver	19,100 pg/g	none	[98]
Methamphetamine	Buffer	9,000 pg/mL	none	[71]
Nicarbazin	Chicken Liver	17,000 pg/g	none	[92]
Nicarbazin	Egg	19,000 pg/g	none	[92]
Norfloxacin	Poultry meat	500 pg/g	none	[84]
Norfloxacin	Egg	1,000 pg/g	none	[84]
Norfloxacin	Fish	1,500 pg/g	none	[84]
Norfloxacin	Fish/Poultry/Egg extracts	1,000–50,000 pg/g	none	[86]
Ochratoxin A	Wine / beverages	58–400 pg/mL	40 nm gold	[12]
Ochratoxin A	Buffer	10 pg/mL	none	[51]
Okadaic Acid	Mussel extract	240 pg/mL	protein label	[55]
Okadaic Acid	Shellfish	20,000 pg/g	none	[56]
Penicillin G	Buffer / milk	1,000–2,000 pg/g	none	[81]
Penicillin G	Milk	2,600 pg/g	none	[89]
Phenytoin	Buffer	1,900 pg/mL	none	[18]
Progesterone	Buffer	8.6 pg/mL	25 nm gold	[4]
Progesterone	Buffer	4.9 pg/mL	10 nm gold	[3]
Progesterone	Milk	400–600 pg/mL	none	[27]
Progesterone	Buffer	35–60 pg/mL	none	[27]
Progesterone	Milk	3,000 pg/mL	none	[28]
Progesterone	Buffer	100 pg/mL	protein label	[15]
Saxitoxin	Shellfish	216,000 pg/g	none	[60]
Stanozolol	Buffer	700 pg/mL	colloidal gold sensor surface	[70]
Staphylococcal enterotoxin	Egg supernatant	1,000 pg/mL	none	[52]
Streptamycin	Milk	20,000 pg/mL	none	[99]
Sulfadiazine	Porcine Bile	28,000 pg/mL	none	[93]
Sulfamethazine	Porcine Bile	15,000 pg/mL	none	[93]
Sulfamethazine	Animal urine	5,000 pg/mL	none	[95]
Sulfamethazine	Milk	1,700 pg/g	none	[96]
Sulfamethazine	Milk	<278 pg/mL	secondary antibody	[97]
T-2/HT-2 toxins	Cereals/baby food	25,000–26,000 pg/g	none	[45]
Testosterone	Saliva	15.4 pg/mL	25 nm gold	[34]
Tetrodotoxin	Buffer	300 pg/mL	none	[64]
Thiamphenicol	Shrimp extract	500 pg/mL	none	[74]
Vitamin B-5	Food extracts	4,400 pg/mL	none	[68]
Warfarin	Plasma ultrafiltrate	4,000 pg/mL	none	[76]

## References

[1] Homola J, Yee SS, Gauglitz G (1999). Surface plasmon resonance sensors: review. Sens. Actuat. B-Chem.

[2] Cooper MA (2002). Optical biosensors in drug discovery. Nat. Rev. Drug Discov.

[3] Yuan J, Oliver R, Li J, Lee J, Aguilar M, Wu Y (2007). Sensitivity enhancement of SPR assay of progesterone based on mixed self-assembled monolayers using nanogold particles. Biosens. Bioeletron.

[4] Mitchell JS, Wu Y, Cook CJ, Main L (2005). Sensitivity enhancement of surface plasmon resonance biosensing of small molecules. Anal. Biochem.

[5] Chung JW, Park JM, Bernhardt R, Pyun JC (2006). Immunosensor with a controlled orientation of antibodies by using NeutrAvidin-protein A complex at immunoaffinity layer. J. Biotechnol.

[6] Lyon LA, Musick MD, Natan MJ (1998). Colloidal Au-enhanced surface plasmon resonance immunosensing. Anal. Chem.

[7] Mitchell JS, Wu Y, Cook CJ, Main L (2006). Estrogen conjugation and antibody binding interactions in surface plasmon resonance biosensing. Steroids.

[8] Mitchell JS, Wu Y (2010). Surface plasmon resonance signal enhancement for immunoassay of small molecules. Methods Mol. Biol.

[9] Jiang XQ, Waterland M, Blackwell L, Wu Y, Jayasundera KP, Partridge A (2009). Sensitive determination of estriol-16-glucuronide using surface plasmon resonance sensing. Steroids.

[10] Jiang XQ, Waterland M, Blackwell L, Partridge A (2010). Determination of estriol-16-glucuronide in human urine with surface plasmon resonance and lateral flow immunoassays. Anal. Method.

[11] Yuan J, Oliver R, Aguilar MI, Wu YQ (2008). Surface plasmon resonance assay for chloramphenicol. Anal. Chem.

[12] Yuan J, Deng DW, Lauren DR, Aguilar MI, Wu YQ (2009). Surface plasmon resonance biosensor for the detection of ochratoxin A in cereals and beverages. Anal. Chim. Acta.

[13] Lyon LA, Pena DJ, Natan MJ (1999). Surface plasmon resonance of Au colloid-modified Au films: particle size dependence. J. Phys. Chem. B.

[14] Driskell JD, Lipert RJ, Porter MD (2006). Labeled gold nanoparticles immobilized at smooth metallic substrates: systematic investigation of surface plasmon resonance and surface-enhanced Raman scattering. J. Phys. Chem. B.

[15] Wu Y, Mitchell J, Cook C, Main L (2002). Evaluation of progesterone-ovalbumin conjugates with different length linkers in enzyme-linked immunosorbent assay and surface plasmon resonance-based immunoassay. Steroids.

[16] Fu E, Nelson KE, Ramsey SA, Foley JO, Helton K, Yager P (2009). Modeling of a competitive microfluidic heterogeneous immunoassay: sensitivity of the assay response to varying system parameters. Anal. Chem.

[17] Foley JO, Nelson KE, Mashadi-Hossein A, Finlayson BA, Yager P (2007). Concentration gradient immunoassay. 2. Computational modeling for analysis and optimization. Anal. Chem.

[18] Nelson KE, Foley JO, Yager P (2007). Concentration gradient immunoassay. 1. An immunoassay based on interdiffusion and surface binding in a microchannel. Anal. Chem.

[19] Moreno-Bondi MC, Taitt CR, Shriver-Lake LC, Ligler FS (2006). Multiplexed measurement of serum antibodies using an array biosensor. Biosens. Bioelectron.

[20] Phillips KS, Wilkop T, Wu JJ, Al-Kaysi RO, Cheng Q (2006). Surface Plasmon resonance imaging analysis of protein-receptor binding in supported membrane arrays on gold substrates with calcinated silicate films. J. Am. Chem. Soc.

[21] Kanoh N, Kyo M, Inamori K, Ando A, Asami A, Nakao A, Osada H (2006). SPR imaging of photo-cross-linked small-molecule arrays on gold. Anal. Chem.

[22] Savchenko A, Kashuba E, Kashuba V, Snopok B (2007). Imaging technique for the screening of protein-protein interactions using scattered light under surface plasmon resonance conditions. Anal. Chem.

[23] Cash KJ, Ricci F, Plaxco KW (2009). A general electrochemical method for label-free screening of protein small molecule interactions. Chem. Commun.

[24] Kawazumi H, Gobi KV, Ogino K, Maeda H, Miura N (2005). Compact surface plasmon resonance (SPR) immunosensor using multichannel for simultaneous detection of small molecule compounds. Sens. Actuat. B-Chem.

[25] Wang JL, Munir A, Zhou HS (2009). Au NPs-aptamer conjugates as a powerful competitive reagent for ultrasensitive detection of small molecules by surface plasmon resonance spectroscopy. Talanta.

[26] Wang JL, Zhou HS (2008). Aptamer-based Au nanoparticles-enhanced surface plasmon resonance detection of small molecules. Anal. Chem.

[27] Gillis EH, Traynor I, Gosling JP, Kane M (2006). Improvement to a surface plasmon resonance-based immunoassay for the steroid hormone progesterone. J. AOAC Int.

[28] Gillis EH, Gosling JP, Sreenan JM, Kane M (2002). Development and validation of a biosensor-based immunoassay for progesterone in bovine milk. J. Immunol. Methods.

[29] Ou HC, Luo ZF, Jiang H, Zhou HM, Wang XP, Song CX (2009). Indirect inhibitive immunoassay for estradiol using surface plasmon resonance coupled to online in-tube SPME. Anal. Lett.

[30] Miyashita M, Shimada T, Miyagawa H, Akamatsu M (2005). Surface plasmon resonance-based immunoassay for 17 beta-estradiol and its application to the measurement of estrogen receptor-binding activity. Anal. Bioanal. Chem.

[31] Thaler M, Metzger J, Schreigg A, Denk B, Gleixner A, Hauptmann H, Luppa P (2005). B. Immunoassay for sex hormone-binding globulin in undiluted serum is influenced by high-molecular-mass aggregates. Clin. Chem.

[32] Kaiser T, Gudat P, Stock W, Pappert G, Grol M, Neumeier D, Luppa PB (2000). Biotinylated steroid derivatives as ligands for biospecific interaction analysis with monoclonal antibodies using immunosensor devices. Anal. Biochem.

[33] Mitchell JS, Lowe TE, Ingram JR (2009). Rapid ultrasensitive measurement of salivary cortisol using nano-linker chemistry coupled with surface plasmon resonance detection. Analyst.

[34] Mitchell JS, Lowe TE (2009). Ultrasensitive detection of testosterone using conjugate linker technology in a nanoparticle-enhanced surface plasmon resonance biosensor. Biosens. Bioelectron.

[35] Ellison PT, Bribiescas RG, Bently GR, Campbell BC, Lipson SF, Panter-Brick C, Hill K (2002). Population variation in age-related decline in male salivary testosterone. Hum. Reprod.

[36] Helton KL, Nelson KE, Fu E, Yager P (2008). Conditioning saliva for use in a microfluidic biosensor. Lab Chip.

[37] Stevens RC, Soelberg SD, Near S, Furlong CE (2008). Detection of cortisol in saliva with a flow-filtered, portable surface plasmon resonance biosensor system. Anal. Chem.

[38] Frasconi M, Mazzarino M, Botre F, Mazzei F (2009). Surface plasmon resonance immunosensor for cortisol and cortisone determination. Anal. Bioanal. Chem.

[39] Sharpe JC, Mitchell JS, Lin L, Sedoglavich N, Blaikie RJ (2008). Gold nanohole array substrates as immunobiosensors. Anal. Chem.

[40] Dillon PP, Daly SJ, Killard AJ, O’Kennedy R (2003). Development and use of antibodies in surface plasmon resonance-based immunosensors for environmental monitoring. Int. J. Environ. An. Ch.

[41] Zhang WW, Chen YC, Luo ZF, Wang JY, Ma DY (2007). Analysis of 17 beta-estradiol from sewage in coastal marine environment by surface plasmon resonance technique. Chem. Res. Chin. U.

[42] Pattnaik P, Srivastav A (2006). Surface plasmon resonance—applications in food science research: A review. J. Food Sci. Tech. Mys.

[43] Homola J (2006). Surface Plasmon resonance (SPR) biosensors and their applications in food safety and security. NATO Sci. Ser. II Math.

[44] Petz M (2009). Recent applications of surface plasmon resonance biosensors for analyzing residues and contaminants in food. Monatsh. Chem.

[45] Meneely JP, Sulyok M, Baumgartner S, Krska R, Elliott CT (2010). A rapid optical immunoassay for the screening of T-2 and HT-2 toxin in cereals and maize-based baby food. Talanta.

[46] Wang XH, Wang S (2008). Sensors and biosensors for the determination of small molecule biological toxins. Sensors.

[47] Hodnik V, Anderluh G (2009). Toxin detection by surface plasmon resonance. Sensors.

[48] Raz SR, Bremer MGEG, Giesbers M, Norde W (2008). Development of a biosensor microarray towards food screening, using imaging surface plasmon resonance. Biosens. Bioeletron.

[49] Pohanka M, Jun D, Kuca K (2007). Mycotoxin assays using biosensor technology: A review. Drug Chem. Toxicol.

[50] Tudos AJ, Lucas-van den Bos ER, Stigter ECA (2003). Rapid surface plasmon resonance-based inhibition assay of deoxynivalenol. J. Agr. Food Chem.

[51] Fu XH (2007). Surface plasmon resonance immunoassay for ochratoxin A based on nanogold hollow balls with dendritic surface. Anal. Lett.

[52] Medina MB (2006). A biosensor method for detection of Staphylococcal enterotoxin A in raw whole egg. J. Rapid Meth. Aut. Mic.

[53] Daly SJ, Keating GJ, Dillon PP, Manning BM, O’Kennedy R, Lee HA, Morgan MRA (2000). Development of surface plasmon resonance-based immunoassay for aflatoxin B-1. J. Agr. Food Chem.

[54] Cuccioloni M, Mozzicafreddo M, Barocci S, Ciuti F, Pecorelli I, Eleuteri AM, Spina M, Fioretti E, Angeletti M (2008). Biosensor-based screening method for the detection of aflatoxins B-1-G(1). Anal. Chem.

[55] Prieto-Simon B, Miyachi H, Karube I, Saiki H (2010). High-sensitive flow-based kinetic exclusion assay for okadaic acid assessment in shellfish samples. Biosens. Bioelectron.

[56] Llamas NM, Stewart L, Fodey T, Higgins HC, Velasco MLR, Botana LM, Elliott CT (2007). Development of a novel immunobiosensor method for the rapid detection of okadaic acid contamination in shellfish extracts. Anal. Bioanal. Chem.

[57] Stevens RC, Soelberg SD, Eberhart BTL, Spencer S, Wekell JC, Chinowsky TM, Trainer VL, Furlong CE (2007). Detection of the toxin domoic acid from clam extracts using a portable surface plasmon resonance biosensor. Harmful Algae.

[58] Traynor IM, Plumpton L, Fodey TL, Higgins C, Elliott CT (2006). Immunobiosensor detection of domoic acid as a screening test in bivalve mollusks: comparison with liquid chromatography-based analysis. J. AOAC Int.

[59] Le Berre M, Kane M (2006). Biosensor-based assay for domoic acid: comparison of performance using polyclonal, monoclonal, and recombinant antibodies. Anal. Lett.

[60] Campbell K, Huet AC, Charlier C, Higgins C, Delahaut p, Elliott CT (2009). Comparison of ELISA and SPR biosensor technology for the detection of paralytic shellfish poisoning toxins. J. Chromatogr. B.

[61] Campbell K, Stewart LD, Douchette GJ, Fodey TL, Haughey SA, Vilarino N, Kawatsu k, Elliott CT (2007). Assessment of specific binding proteins suitable for the detection of paralytic shellfish poisons using optical biosensor technology. Anal. Chem.

[62] Tsumoto K, Yokota A, Tanaka Y, Ui M, Tsumuraya T, Fujii I, Kumagai I, Nagumo Y, Oguri H, Inoue M, Hirama M (2008). Critical contribution of aromatic rings to specific recognition of polyether rings – the case of ciguatoxin CTX3C-ABC and its specific antibody 1C49. J. Biol. Chem.

[63] Oguri H (2007). Bioorganic studies utilizing rationally designed synthetic molecules: absolute configuration of ciguatoxin and development of immunoassay systems. B. Chem. Soc. Jpn.

[64] Taylor AD, Ladd J, Etheridge S, Deeds J, Hall S, Jiang SY (2008). Quantitative detection of tetrodotoxin (TTX) by a surface plasmon resonance (SPR) sensor. Sens. Actuat.-B Chem.

[65] Kim SJ, Gobi KV, Iwasaka H, Tanaka H, Miura N (2007). Novel miniature SPR immunosensor equipped with all-in-one multi-microchannel sensor chip for detecting low-molecular-weight analytes. Biosens. Bioelectron.

[66] Kim SJ, Gobi KV, Tanaka H, Shoyama Y, Miura N (2006). Enhanced sensitivity of a surface-plasmon-resonance (SPR) sensor for 2,4-D by controlled functionalization of self-assembled monolayer-based immunosensor chip. Chem. Lett.

[67] Nabok AV, Tsargorodskaya A, Holloway A, Starodub NF, Gojster O (2007). Registration of T-2 mycotoxin with total internal reflection ellipsometry and QCM impedance methods. Biosens. Bioelectron.

[68] Haughey SA, O’Kane AA, Baxter CA, Kalman A, Trisconi MJ, Indyk HE, Watene GA (2005). Determination of pantothenic acid in foods by optical biosensor immunoassay. J. AOAC Int.

[69] Kreuzer MP, Quidant R, Badenes G, Marco MP (2006). Quantitative detection of doping substances by a localized surface plasmon sensor. Biosens. Bioelectron.

[70] Kreuzer MP, Quidant R, Salvador JP, Marco MP, Badenes G (2008). Colloidal-based localized surface plasmon resonance (LSPR) biosensor for the quantitative determination of stanozolol. Anal. Bioanal. Chem.

[71] Smith JP, Martin A, Sammons DL, Striley C, Biagnini R, Quinn J, Cope R, Snawder JE (2009). Measurement of methamphetamine on surfaces using surface plasmon resonance. Toxicol. Mech. Method.

[72] Klenkar G, Liedberg B (2008). A microarray chip for label-free detection of narcotics. Anal. Bioanal. Chem.

[73] Johansson MA, Hellenas KE (2004). Matrix effects in immunobiosensor determination of clenbuterol in urine and serum. Analyst.

[74] Dumont V, Huet AC, Traynor I, Elliott C, Delahaut P (2006). A surface plasmon resonance biosensor assay for the simultaneous determination of thiamphenicol, florefenicol, florefenicol amine and chloramphenicol residues in shrimps. Anal. Chim. Acta.

[75] Gaudin V, Maris P (2001). Development of a biosensor-based immunoassay for screening of chloramphenicol residues in milk. Food Agr. Immunol.

[76] Fitzpatrick B, O’Kennedy R (2004). The development and application of a surface plasmon resonance-based inhibition immunoassay for the determination of warfarin in plasma ultrafiltrate. J. Immunol. Meth.

[77] Lofgren JA, Dhandapuni S, Pennucci JJ, Abbott CM, Mytych DT, Kaliyaperumal A, Swanson SJ, Mullenix MC (2007). Comparing ELISA and surface plasmon resonance for assessing clinical immunogenicity of panitumumab. J. Immunol.

[78] Moghaddam A, Borgen T, Stacy J, Kausmally L, Simonsen B, Marvik OJ, Brekke OH, Braunagel M (2003). Identification of scFv antibody fragments that specifically recognize the heroin metabolite 6-monoacetylmorphine but not morphine. J. Immunol. Methods.

[79] Blasco C, Torres CM, Pico Y (2007). Progress in antibacterials analysis of residual in food. Trac-Trend Anal. Chem.

[80] Davis F, Higson SPJ (2010). Label-free immunochemistry approach to detect and identify antibiotics in milk. Pediatr. Res.

[81] Gustavsson E, Bjurling P, Degelaen J, Sternesjo A (2002). Analysis of beta-lactam antibiotics using a microbial receptor protein-based biosensor assay. Food Agric. Immunol.

[82] Keegan J, Whelan M, Danaher M, Crooks S, Sayers R, Anastasio A, Elliott C, Brandon D, Furey A, O’Kennedy R (2009). Benimidazole carbamate residues in milk: detection by surface plasmon resonance-biosensor, using a modified QuEChERS (Quick, Easy, Cheap, Effective, Rugged and Safe) method for extraction. Anal. Chim. Acta.

[83] Raz SR, Bremer MGEG, Haasnoot W, Norde W (2009). Label-free and multiplex detection of antibiotic residues in milk using imaging surface plasmon resonance-based immunosensor. Anal. Chem.

[84] Huet AC, Charlier C, Weigel S, Godefroy SB, Delahaut P (2009). Validation of an optical surface plasmon resonance biosensor assay for screening (fluoro)quinolones in egg, fish and poultry. Food Addit. Contam. A.

[85] Huet AC, Charlier C, Singh G, Godefroy SB, Leivo J, Vehniaeinen M, Nielen MWF, Weigel S, Delahaut P (2008). Development of an optical surface plasmon resonance biosensor assay for (fluoro) quinolones in egg, fish and poultry meat. Anal. Chim. Acta.

[86] Weigel S, Pikkemaat MG, Elferink JWA, Mulder PPJ, Huet AC, Delahaut P, Schittko S, Flerus R, Nielen M (2009). Comparison of a fluoroquinolone surface plasmon resonance biosensor screening assay with established methods. Food Addit. Contam. A.

[87] Haasnoot W, Gercek H, Cazemier G, Nielen MWF (2007). Biosensor immunoassay for flumequine in broiler serum and muscle. Anal. Chim. Acta.

[88] Marchesini GR, Haasnoot W, Delahaut P, Gerseck H, Nielen MWF (2007). Dual biosensor immunoassay-directed identification of fluoroquinolones in chicken muscle by liquid chromatography electrospray time-of-flight mass spectrometry. Anal. Chim. Acta.

[89] Gustavsson E, Bjurling P, Sternesjo A (2002). Biosensor analysis of penicillin G in milk based on the inhibition of carboxypeptidase activity. Anal. Chim. Acta.

[90] Dillon P, Daly S, Browne J, Manning B, O’Kennedy R, van Amerongen A (2003). Development of surface plasmon resonance-based immunoassay for cephalexin. P-Soc. Photo-Opt. Ins.

[91] Dillon PP, Daly SJ, Browne JG, Manning BM, Loomans E, van Amerongen A, O’Kennedy R (2003). Application of an immunosensor for the detection of the beta-lactam antibiotic, cephalexin. Food Agr. Immunol.

[92] McCarney B, Traynor IM, Fodey TL, Crooks SRH, Elliott CT (2003). Surface plasmon resonance biosensor screening of poultry liver and eggs for nicarbazin residues. Anal. Chim. Acta.

[93] Situ C, Crooks SRH, Baxter AG, Ferguson J, Elliott CT (2002). On-line detection of sulfamethazine and sulfadiazine in porcine bile using a multi-channel high-throughput SPR biosensor. Anal. Chim. Acta.

[94] Crooks SRH, Stenberg E, Johansson MA, Hellenas KE, Elliott CT (2001). The use of optical biosensor for high-throughput detection of veterinary drug residues in foods. P. Soc. Photo.-Opt. Ins.

[95] Akkoyun A, Kohen VF, Biltewski U (2000). Detection of sulphamethazine with an optical biosensor and anti-idiotypic antibodies. Sens. Actuat. B-Chem.

[96] Gaudin V, Pavy ML (1999). Determination of sulfamethazine in milk by biosensor immunoassay. J. AOAC Int.

[97] Sternesjo A, Mellgren C, Bjorck L (1995). Determination of sulfamethazine residues in milk by a surface-plasmon resonance-based biosensor assay. Anal. Biochem.

[98] Samsonova JV, Baxter GA, Grooks SRH, Small AE, Elliott CT (2002). Determination of ivermectin in bovine liver by optical immunobiosensor. Biosens. Bioelectron.

[99] Haasnoot W, Loomans EEMG, Cazemier G, Dietrich R, Verheijen R, Bergwerff AA, Stephany RW (2002). Direct *versus* competitive biosensor immunoassays for the detection of (dihydro)streptomycin residues in milk. Food Agr. Immunol.

[100] Keating GJ, Quinn JG, O’Kennedy R (1999). Immunoassay for the determination of 7-hydroxycoumarin in serum using ‘real-time’ biosensor analysis. Anal. Lett.

[101] Smith RG, D’Souza N, Nicklin S (2008). A review of biosensors and biologically-inspired systems for explosives detection. Analyst.

[102] Shankaran DR, Kawaguchi DR, Kim SJ, Matsumoto K, Toko K, Miura N (2006). Evaluation of the molecular recognition of monoclonal and polyclonal antibodies for sensitive detection of 2,4,6-trinitortoluene (TNT) by indirect competitive surface plasmon resonance immunoassay. Anal. Bioanal. Chem.

[103] Singh P, Onodera T, Mizuta Y, Matsumoto K, Miura N, Toko K (2009). Dendrimer modified biochip for detection of 2,4,6-trinitrotoluene on SPR immunosensor: fabrication and advantages. Sens. Actuat.-B Chem.

[104] Larsson A, Angbrant J, Ekeroth J, Mansson P, Liedberg B (2006). A novel biochip technology for detection of explosives—TNT: synthesis, characterization and application. Sens. Actuat.-B Chem.

[105] Bowen J, Noe LJ, Sullivan BP, Morris K, Martin V, Donnelly G (2003). Gas-phase detection of trinitrotoluene utilizing a solid-phase antibody immobilized on a gold film by means of surface plasmon resonance spectroscopy. Appl. Spectrosc.

[106] Riskin M, Tel-Vered R, Willner I (2010). Imprinted Au-nanoparticle composites for the ultrasensitive surface plasmon resonance detection of hexahydro-1,3,5-trinitro-1,3,5-triazine (RDX). Adv. Mater.

[107] Nagatomo K, Kawaguchi T, Miura N, Toko K, Matsumoto K (2009). Development of a sensitive surface plasmon resonance immunosensor for detection of 2,4-dinitrotoluene with a novel oligo(ethylene glycol)-based sensor surface. Talanta.

